# Revision of meso-Rex bypass utilizing a collateral vein in a patient with portal steal phenomenon after liver transplant: A case report

**DOI:** 10.1016/j.ijscr.2019.05.051

**Published:** 2019-06-04

**Authors:** Ruben Blachman-Braun, Fidel Lopez-Verdugo, Diane Alonso, Linda Book, G. Peter Feola, Manuel I. Rodriguez-Davalos

**Affiliations:** a Pediatric Liver Transplantation, Intermountain Primary Children’s Hospital, Salt Lake City, UT, USA; b Pediatric Liver Disease and Transplant Program, Intermountain Primary Children’s Hospital, Department of Pediatrics, University of Utah School of Medicine, Salt Lake City, UT, USA; c Department of Radiology, Intermountain Primary Children's Hospital, Salt Lake City, UT, USA

**Keywords:** Meso-Rex bypass, Collateral vein, Pediatric liver transplantation, Portal steal phenomenon

## Abstract

**Introduction:**

In children with extrahepatic portal vein obstruction or those who develop portal vein thrombosis after liver transplant, the use of Meso-Rex Bypass (MRB) creates a more physiological state by redirecting mesenteric blood flow back into the intrahepatic portal system via a venous conduit.

**Presentation of case:**

A 3-year-old female with biliary atresia associated with polysplenia syndrome and a surgical history of Kasai portoenterostomy procedure, and an ABO incompatible whole liver transplant. Within a year after transplant she presented with prehepatic portal hypertension, that was treated with MRB using a deceased donor ABO compatible iliac vein as conduit. Six months later, she was taken to the operating room for bypass revision, during the procedure the MRB showed no flow and no thrombus, and a large splenorenal collateral vein that was causing a portal perfusion steal phenomenon was observed. After dissecting the collateral vein, an 8 cm x8 mm segment of this vessel was used as an autologous conduit to re-do the Rex.

**Discussion:**

Failed of MRB can be attributed to portal steal phenomenon, hypercoagulable disorders, bypass contraction or kinking. In this case we believe the culprit to be the former. When there is a history of longstanding portal hypertension, large collaterals develop; thus, intraoperative portal vein flow measurement is critical and ligation of large collaterals during liver transplantation and MRB should be performed to avoid portal steal phenomenon postprocedure.

**Conclusion:**

Using a collateral vein as an alternative autologous venous conduit is a feasible option that can have durable success.

## Highlights

• Re-do of a meso-Rex Bypass is a feasible option with good outcomes.
• A large collateral vein is an alternative for an autologous venous conduit in a MRB.
• Large collaterals ligation during MRB should be performed to avoid portal steal.

## 1 Introduction

Extrahepatic portal vein obstruction (EHPVO) is a chronic entity characterized by obstruction of the extrahepatic portal vein with or without the involvement of the intrahepatic portal veins that leads to prehepatic portal hypertension, cavernoma formation, and dilation of portosystemic collateral veins [1,2]. After liver transplantation, it is estimated that 1–8.5% of the pediatric patients will develop portal vein complications, in the case of portal vein thrombosis (PVT) it can jeopardize short and long-term outcomes of the transplant [3,4]. Thus, in children with prehepatic portal hypertension secondary to PVT, a meso-Rex bypass (MRB) procedure should be considered to redirect the portal blood flow into the intrahepatic portal system [5,6].

MRB has shown to substantially improve portal hypertension. Though, as any other procedure MRB is not exempt from complications. Failure of the MRB due to occlusion has been reported to occur in 10% [7,8] to 40% of patients [9,10] at a 6-month follow-up. Early thrombosis leading to MRB failure is believed to be related to a variety of factors, including an inadequate graft type for bypass creation, undiagnosed hypercoagulable disorders that can lead to intraluminal thrombus formation, poor patient selection (i.e., those with a primitive intrahepatic portal venous system) [11], bypass contraction or kinking that decreases the intraluminal area, and portal perfusion steal phenomenon, among others. Cornerstones of management of failed MRB includes anticoagulation, endovascular intervention, direct thrombectomy, revision and re-do of MRB, or conversion to a portosystemic shunt (PSS) [12].

Herein, we present a case report following the SCARE guidelines [13], of a patient with biliary atresia associated with polysplenia syndrome (BA-PS) that underwent liver transplantation and subsequent MRB, then developed a portal steal phenomenon due to a large collateral vein. During the revision of the bypass we report an innovative approach in which the collateral vein that was causing the diversion of the portal flow was used to create an autologous venous conduit for the re-do MRB.

## 2 Presentation of case

A 3-year-old female with a diagnosis of BA-PS had a Kasai portoenterostomy procedure at 12 weeks of age. Due to the progression of her liver disease, she was listed for transplant at the age of 6 months with a Pediatric End-Stage Liver Disease (PELD) of 13. Four months later she underwent an orthotopic liver transplant with a whole graft from an ABO incompatible 7-month-old deceased donor (DD). Within a year of transplant, the patient developed symptoms of prehepatic portal hypertension secondary to PVT and was referred to our team for surgical consultation. Fourteen months after transplantation we performed a mesenteric to left portal vein bypass also known as MRB. The bypass was done using an ABO compatible DD iliac vein. During surgery, a spontaneous portosystemic shunt was identified and the decision at the time was not to ligate since the intraoperative Doppler demonstrated velocities ranging between 30 and 50 cm/s. The patient had an uncomplicated post-surgical course and repeat Doppler ultrasounds at 1 week (52 cm/s) and 1-month (16.5 cm/s) post-MRB demonstrated continued left portal vein flow.

**Fig. 1:**
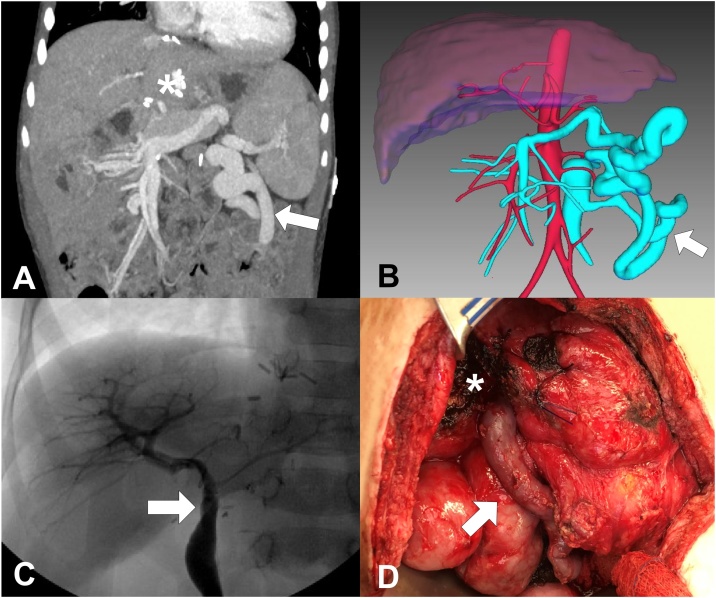
**A)** CT scan before the revision of the MRB showing a large splenorenal collateral vein (arrow), and the Rex recess (*). **B)** 3D reconstruction before the revision of the MRB showing a large splenorenal collateral vein (arrow). **C)** Intraoperative portogram performed during the revision of the MRB (arrow), showing a normal intrahepatic portal system. **D)** Image showing the re-do MRB (arrow) that was performed using a splenorenal collateral vein as an autologous venous conduit, Rex recess (*).

**Fig. 2:**
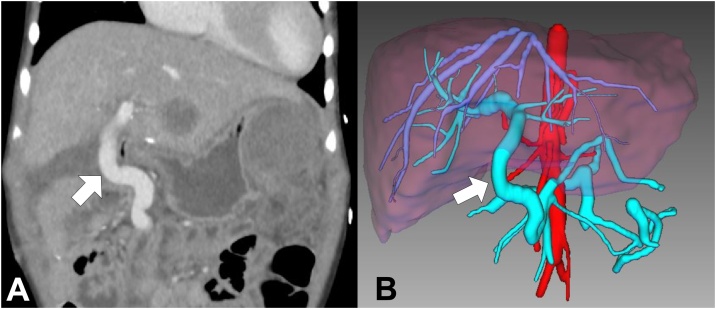
**A)** CT scan 1 month after the re-do MRB showing a patent conduit (arrow). **B)** 3D reconstruction of the re-do MRB 1 month after the operation.

Six months after the initial MRB the patient again developed symptoms of portal hypertension, splenomegaly with platelets of 89,000/mcL and a serum ammonia of 101 mcmol/L. We hypothesized that a large splenorenal collateral vein was potentially causing a portal venous perfusion steal phenomenon (Fig. 1A and B). Therefore, 10 months after the initial MRB, the patient was taken to the operating room for revision of the MRB. Prior to surgery, the proposed plan was to take down the collateral and attempt a re-do MRB using the patient’s internal jugular or convert to a splenorenal shunt. During surgery, the MRB was found patent and collapsed with no flow in the presence of a dominant left sided collateral vein. In the process of taking down this collateral to mitigate the steal we realized that this vessel was potentially suitable for an autologous venous conduit. This collateral was dissected to create an 8 cm × 8 mm autologous vein conduit that was used to revise the previous bypass. This was anastomosed to the left portal vein at the Rex recess and a portogram demonstrated adequate intrahepatic portal system (Fig. 1C), followed by anastomosis to the spleno-mesenteric junction (Fig. 1D). A CT scan was performed 1 week after surgery demonstrating conduit patency. Then, a Doppler ultrasound which demonstrated continued adequate flow within the bypass (40 cm/s) 1 month after re-do MRB. On follow-up, CT scan demonstrated sustained patency at 1- (Fig. 2) and 6-month follow up. Serum ammonia (34 mcmol/L), and platelets (314,000/mcL) were within normal limits six months after re-do MRB.

## 3 Discussion

It is estimated that the biliary atresia prevalence ranges from 0.5 to 2.6 per 10,000 live birth, and the variability of biliary atresia prevalence globally has been attributed to different ethnical, genetic, and environmental factors. Biliary atresia can present as an isolated finding, in combination with other congenital anomalies, or as part of a syndrome, within the former clinical presentation the BA-PS has been described [14].

Children with BA-PS have an inflammatory cholangiopathy that can lead to fibrosis, and biliary cirrhosis, causing portal hypertension. Thus, the development of portosystemic collaterals (i.e., spontaneous shunts) is frequently seen in these cases. Although the portal vein resistance decreases after transplant, shunts might remain open allowing for the non-physiological diversion of the portal venous flow. In addition, the typical anastomosis between the donor portal vein to an often atretic or hypoplastic portal vein seen in biliary atresia has a higher risk of thrombosis [6,[15], [16], [17]].

**Fig. 3:**
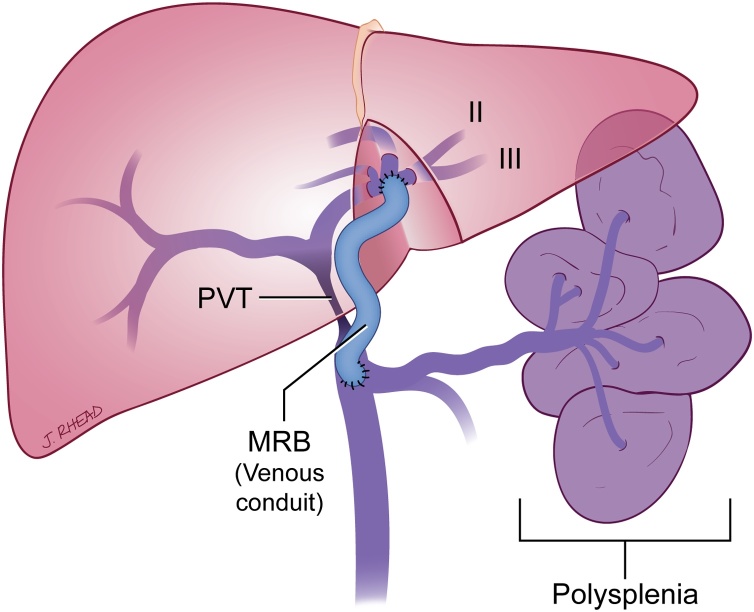
Drawing of a MRB in a patient with and biliary atresia associated with polysplenia syndrome and portal vein thrombosis (PVT) after transplant.

In the past when conservative management of children with portal hypertension failed, selective PSS were performed. However, in the last decade the use of MRB has increased in popularity as it creates a more physiological state by redirecting the mesenteric blood flow back into the liver (Fig. 3) [18]. This is accomplished by using a conduit to connect the mesenteric venous system to the left portal vein in the space of Rex. This effectively allows for access to the lower resistance intrahepatic portal system and avoids the hepato-fugal nature of surgical portosystemic shunts [6,15]. Although both MRB and PSS have proven to be effective in relieving portal hypertension, evidence suggests that MRB have the additional benefit of improvement in platelet count, international normalized ratio (INR), and somatic growth [18].

Autologous venous conduits are preferred in MRB, with the most common conduit being the internal jugular vein, however other options such as an autogenous saphenous vein, splenic vein, right gastroepiploic vein, inferior mesenteric vein or umbilical vein have been described in the literature [6,19]. In this case, after clinical suspicion of MRB failure, a decision was made to perform a revision and re-do using an autologous internal jugular vein, the rationale being that the DD allograft led to an increased risk of thrombosis. However, the absence of a thrombus inside the conduit prompted us to consider a portal steal phenomenon as the main cause of MRB failure.

At times, the capacity of a primitive intrahepatic portal venous system will not suffice to accommodate the increased blood flow immediately after MRB, redirecting blood flow back into the low-pressure system of large collateral vessels. Failure to ligate portosystemic collaterals at the time of surgery will not only significantly decrease the amount of blood flow redirected to the liver, depriving the patient from the additional benefits of hepatopetal flow restoration, but will also fail to address the main goal of surgical treatment, which is to decrease the risk of future gastroesophageal variceal bleeding episodes. Therefore, our group now favors taking down collaterals at the time of liver transplant or MRB to avoid the development of portal steal phenomenon and subsequent thrombosis [15].

## 4 Conclusion

This case reports the option of using a large collateral vein as an alternative for an autologous venous conduit in a MRB and demonstrates that re-do of a MRB is a feasible option with good outcomes. In addition, in children with longstanding portal hypertension ligation of large collaterals during liver transplantation or MRB should be performed to avoid portal steal phenomenon post-procedure.

## Conflict of interest

None.

## Sources of funding

None.

## Ethical approval

This is a case report and is exempt for ethical approval at the authors institution.

## Consent

Written informed consent was obtained from the mother of the patient for publication of this case report and accompanying images. A copy of the written consent is available for review by the Editor-in-Chief of this journal on request.

## Author contribution

RBB, FLV, DA, LB, GPF, and MIRD did significantly contribute to the conception or design of the work, the acquisition, and interpretation of data/findings. All authors have participated sufficiently in the work to take public responsibility for appropriate portions of the content. All authors critically revised the final manuscript. All authors approved the final version of the manuscript to be published.

## Registration of research studies

Not applicable.

## Guarantor

Ruben Blachman-Braun.

## Provenance and peer review

Not commissioned, externally peer-reviewed.
